# The association between diabetes and age at the onset of menopause: a systematic review protocol

**DOI:** 10.1186/s13643-019-0989-5

**Published:** 2019-04-02

**Authors:** Mansoureh Yazdkhasti, Zahra Mehdizadeh Tourzani, Nasibeh Roozbeh, Vajiheh Hasanpour, Sara Esmaelzadeh Saeieh, Fatemeh Abdi

**Affiliations:** 1 Department of Midwifery, Faculty of Midwifery, Social Determinations of Health Research Center, Alborz University of Medical Sciences, Karaj, Iran; 2 Department of Midwifery, Faculty of Midwifery, Alborz University of Medical Sciences, Karaj, Iran; 3 Reproductive Health, Mother and Child Welfare Research Center, Hormozgan Universiy of Medical Sciences, BandarAbbas, Iran; 4 Department of Midwifery, Nursing and Midwifery Faculty, Shahid Beheshti University of Medical Sciences, Tehran, Iran; 5 Student Research Committee, Nursing and Midwifery Faculty, Shahid Beheshti University of Medical Sciences, Tehran, Iran

**Keywords:** Menopausal age, Diabetes, DM I/II

## Abstract

**Background:**

Age at the onset of menopause is the most important determinant of women’s future health outcomes. While the basic mechanisms contributing to the onset of menopause are still not fully understood, age at menopause depends on a complex set of various factors. In this regard, the effects of diabetes (DM I/II) on the age at the onset of menopause have received little attention.

**Methods and analysis:**

Electronic databases including PubMed/MEDLINE, Web of Science, Scopus, EMBASE, and Google Scholar will be searched for articles published during January 2000 to August 2018 and containing combinations of related MeSH terms, i.e., “age at menopause” and “diabetes.” Additional studies will also be extracted from the reference lists of the selected papers, gray literature, and key journals in the field. A set of inclusion criteria will be defined, and all eligible observational studies will be included. Two reviewers will independently conduct the study selection, data extraction, and quality assessment of the selected studies. All cases of disagreement will be resolved through consensus. The methodological assessment of the primary studies will be performed based on the Newcastle-Ottawa Scale (NOS). In case of the availability of sufficient data, fixed or random effects models will be used to combine all data. Heterogeneity will be assessed by *I*^2^ statistic and chi-square test. Stata V.11.1 will be used for data analysis (CRD42017080789).

**Ethics and dissemination:**

This systematic review will not raise any ethical issues. Journal publication and conference presentations will facilitate the wide dissemination of the findings to relevant clinicians and researchers.

## Background

**Fig. 1:**
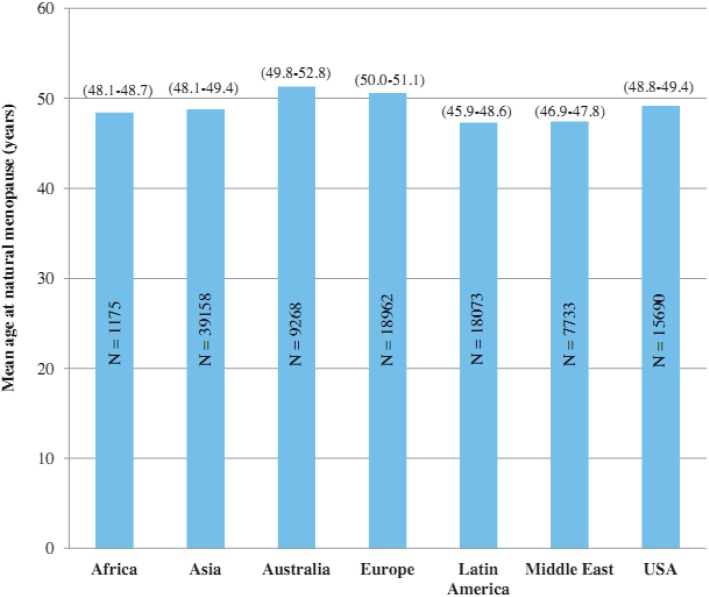
Mean age at natural menopause (95% confidence interval) in different parts of the world

Menopause, defined as the absence of menstruation periods for at least 12 months [1], is a multidimensional evolutionary process with major effects on women’s quality of life and risk of developing particular diseases [2, 3]. Age at the onset of menopause is the most important determinant of women’s future health outcomes. While the exact mechanisms affecting the onset of menopause are still not fully understood, due to a complex combination of factors, age at menopause is different in different parts of the world [4]. In Western women, menopause typically occurs between 40 and 60 years of age (average age 51 years) [5]. However, women’s average menopause age differs in Asia, Africa, Australia, and the Middle East (Fig. 1) [6].

The World Health Organization (WHO) has estimated the number of postmenopausal women worldwide to reach 1.2 billion by 2030. A total of 47 million women go through menopause each year [7]. Age at natural menopause (i.e., the last natural menstruation) can largely affect the risk of both morbidity and mortality [8]. Age at natural menopause may vary depending on a number of demographic, socioeconomic, cultural, reproductive, and lifestyle-related factors [9, 10]. On the other hand, it can alter the risk of various health conditions including hypertension, diabetes, gastroenteritis, chronic renal disease, and cardiovascular diseases [11, 12]. Meanwhile, certain chronic diseases (e.g., diabetes) seem to accelerate reproductive aging and possibly lead to more premature ovarian aging [13].

According to the European Prospective Investigation, women who developed diabetes at a young age (< 20 years) had a lower age at the onset of menopause. In contrast, developing diabetes at an older age (> 50 years) was associated with delayed menopause [14]. The relationship between diabetes and early menopause was also confirmed by another study on women younger than 45 years old [5].

Type 1 diabetes causes premature vascular aging which in turn leads to ovarian aging and early menopause. Based on a comparison between women with type 1 diabetes and women without diabetes, the risk of early depletion of the ovarian follicle pool in diabetic women increased their chances of earlier menopause at a younger age. Moreover, compared to their non-diabetic counterparts, women with type 1 diabetes have a higher age at menarche and are more likely to experience menstrual irregularities. These women’s delayed age at menarche and earlier onset of menopause decreases their reproductive period by about 6 years [15].

Women who experience premature menopause (before age 40 years) or early menopause (between ages 40 and 45 years) will increase the risk of early development of complications. Therefore, preventing early menopause through prevention/control of DM I/II can serve as an effective measure to improve women’s health and reduce health care costs. To the best of our knowledge, most systematic reviews have focused on the effects of menopause on DM I/II. Few studies have investigated the impacts of DM I/II on age at the onset of menopause. Also, there are different conclusions between the various studies that whether DM I/II effect on age at the onset of menopause or not. Therefore, this systematic review will explore the association between DM I/II and age at the onset of menopause.

## Objectives

The primary goal of this study will be to clarify whether or not women suffering from DM I/II before the onset of menopause would experience an earlier menopause. In other words, this study will seek to determine the association between DM I/II and age at the onset of menopause. A secondary objective in this study will be to investigate the heterogeneity of previous studies.

## Methods

### Registration and methodology

The study protocol was registered in the International Prospective Register of Systematic Reviews (PROSPERO) at the National Institute for Health Research. Registration number in PROSPERO is CRD42017080789. The guidelines of PRISMA-P (Preferred Reporting Items for Systematic Review and Meta-Analysis Protocols) were followed while reporting the study protocol.

### Eligibility criteria

Observational studies (cross-sectional, cohort, and case-control studies) evaluating the relationship of premature menopause (under 40 years of age), early menopause (between ages 40 and 45 years), premature ovarian failure (POF) (under 40 years of age), or menopause at a normal age with type I/II diabetes mellitus are included in the criteria.

### Exclusion criteria

Non-observational primary studies (qualitative and interventional studies, i.e., CT/RCT), studies on women with surgical menopause (hysterectomy + bilateral salpingo-oophorectomy (BSO)) or BSO, studies on menopausal women with various types of cancers, studies on radiotherapy or chemotherapy, induced menopause, studies on menopausal women with drug abuse, and studies on menopausal women undergoing hormone therapy will be excluded.

### Types of studies

All observational studies (cross-sectional/analytical, population-based cohort studies or cohort studies on particular groups, prospective or retrospective cohort studies, and case-control or nested cohort studies) will be included in this systematic review. Studies conducted on qualitative and interventional studies, i.e., CT/RCT, will not be included. All studies should address the relationship between diabetes and age at menopause. No language restriction will be applied.

### Participants

This systematic review will recruit adult women (women over 18 years of age who had their menarche) with an experience of type I/II diabetes before menopause. Women who developed the mentioned conditions after menopause will not be included. Menopause is defined as the absence of menstrual periods for at least 1 year (12 full months). The average age of menopause is 51 years, but varies among different individuals and populations. Early (between ages 40 and 45 years) and premature (before age 40 years) menopause refers to menopause, respectively. All women with natural menopause will be included regardless of age at menopause. Women’s age at menopause will be determined by self-report or laboratory criterion, i.e., follicle-stimulating hormone (FSH) levels over 30 mIU/mL. Since women with natural, not induced, menopause will be of interest, those with surgical menopause (following hysterectomy accompanied by bilateral oophorectomy) or menopause induced by certain medications (e.g., chemotherapy drugs) or radiotherapy will not be included. Moreover, women with drug or alcohol abuse and those receiving hormone therapy will be excluded from this study.

### Primary outcomes

This systematic review will evaluate observational-analytical studies which focus on the associations of menopause age with type I/II diabetes, hypertension, or atherosclerosis as their primary outcomes.

### Secondary outcome

The methodological and statistical heterogeneity assessment, along with the sensitivity analysis, of the selected studies will be the secondary outcomes of this systematic review.

### Search strategy

A literature search will be performed according to the Preferred Reporting Items for Systematic review and Meta-Analysis Protocols (PRISMA-P) guidelines. No language restrictions will be imposed during the search.

Articles published in well-known databases including PubMed/MEDLINE, Web of Science, Scopus, EMBASE, and Google Scholar (up to five pages) during January 1, 2000, to August 1, 2018, will be searched. “Age at menopause” and “diabetes” will be used as key terms during the search process. Table 1 presents the search strategy applicable to finding relevant studies containing “Diabetes” AND “Menopause” in PubMed/MEDLINE. The same search strategy, of course with the required modifications, will be used for other electronic databases.

**Table 1:** Search strategy used to extract articles from PubMed

1	(Diabetes Mellitus AND Noninsulin Dependent) OR (Diabetes Mellitus AND Ketosis Resistant) OR (Ketosis-Resistant Diabetes Mellitus) OR (Diabetes Mellitus AND Non Insulin Dependent) OR (Non-Insulin-Dependent Diabetes Mellitus) OR (Diabetes Mellitus AND Stable) OR (Stable Diabetes Mellitus) OR (Diabetes Mellitus AND Type II) OR (NIDDM) OR (Diabetes Mellitus AND Noninsulin Dependent) OR (Diabetes Mellitus AND Maturity-Onset) (Postmenopausal) OR (PostMenopause) OR (Post-menopausal Period) AND (Cohort study)AND(Diabetes Mellitus AND Noninsulin Dependent) OR (Diabetes Mellitus AND Ketosis Resistant) OR (Ketosis-Resistant Diabetes Mellitus) OR (Non-Insulin-Dependent Diabetes Mellitus) OR (Diabetes Mellitus AND Stable) OR (Stable Diabetes Mellitus) OR (Diabetes Mellitus AND Type II) OR (NIDDM) OR (Diabetes Mellitus AND Noninsulin Dependent) OR (Diabetes Mellitus AND Maturity-Onset)
1#2	AND (Cohort study) AND (early menopause)

### Other resources

Additional studies will be extracted from not only the reference lists of the selected articles, but also key journals and gray literature in the field.

### Data collection and analysis

After entering the extracted documents into an EndNote (X6) library and removing the duplicates, the title and abstracts of the remaining papers will be evaluated in terms of relevance and irrelevant articles will be excluded. Two reviewers will then independently assess the eligibility of the full texts of relevant studies based on the inclusion and exclusion criteria. Any cases of disagreement between the two reviewers will be resolved through consensus.

After identifying eligible articles, two reviewers will independently extract the required data including study characteristics (i.e., the first author’s name and the setting, design, and publication year of the study), menopause-related data including age at menopause and type of menopause (premature, early, or natural), participant characteristics (i.e., age and ethnicity), and key measures (i.e., mean ± SD, relative risk (RR), the crude odds ratio (OR) and adjusted OR, 95% CI (lower limit-upper limit), sample size (*N*), mean difference, and standard mean difference). Cohen’s *d* is determined by calculating the mean difference between two groups, and then dividing the result by the pooled standard deviation (Cohen’s *d* = (*M*_2_ − *M*_1_)/*SD*_poole_*)*; mean (*M*); standard deviation (*SD*); sample size (*n*)) via Web plot Digitizer. The confidence interval (CI) is used for the standard error (SE).

All publications reporting similar findings of a single study will be regarded as one study. The authors of potentially relevant unpublished papers will also be contacted three times with 15-day intervals (if no response is received the first time). The studies will be excluded if their authors fail to respond. All cases of disagreement between the two reviewers will be resolved through consensus.

### Assessment of methodological quality

Two researchers will independently evaluate the methodology of preliminary studies based on the Newcastle-Ottawa Scale (NOS). The NOS measures the risk of bias in various observational studies, including cross-sectional, cohort, and case-control studies, and scores the least risk of bias in three domains, i.e., selection of study groups, comparability of groups, and ascertainment of exposure and outcomes, to a maximum of 9 points [16].

### Statistical analysis and data synthesis

All extracted data will be analyzed using Stata V.11.1 (StataCorp LP, College Station, TX, USA). The association between DM I/II and age at the onset of menopause will be evaluated through meta-analysis. If meta-analysis is not feasible, narrative synthesis may be conducted (for example, results organized by major outcomes and, within this, will be presented separately for each comparison) [17]. The effect estimates (mean difference) and their standard deviation from each study will be combined with a fixed effects or random effects model. Because statistically significant (*P* values) is influenced by the sample size, so statistically significant and clinically important are used. Moreover, the relative risk of early menopause in women with DM I/II will be determined compared to those without DM I/II.

### Heterogeneity assessment

Methodological and statistical heterogeneities will be assessed using the *I*^2^ statistic and chi-square test, respectively. There is no uniform approach to dealing with heterogeneity. Multiple strategies have been proposed; subgroup analysis or meta-regression will be applied in case of heterogeneity. Cochran’s *Q* is used for the assessment of heterogeneity in meta-analysis. The classical measure of heterogeneity is Cochran’s *Q*, which is calculated as the weighted sum of squared differences between individual study effects and the pooled effect across studies, with the weights being those used in the pooling method. The existing guideline will be used to interpret the *I*^2^ statistic. In cases of substantial heterogeneity (*I*^2^ > 50%) between the studies, the results will be qualitatively described and not pooled. In addition, methodological quality assessment suggests the dissimilarity of the preliminary studies [18]. Sources of heterogeneity can be different menopause ages and type I/II diabetes, ethnicity, and methods for diagnosis of diabetes. Methodological heterogeneity will be evaluated after assessing the methodological quality of preliminary studies and the fixed or random effects models (FEM or REM, respectively) will be developed accordingly. The decision to use a model for the combination of preliminary studies is not made based on statistical heterogeneity alone. A FEM will be selected if the quality assessment of preliminary studies confirms their similarity in methodology and difference in sample size. A REM, which is a more conservative method, will be used if methodological quality assessment suggests the dissimilarity of the preliminary studies. Subgroup analysis or meta-regression may be applied by the authors.

Authors formally rate or assess the overall body of evidence addressed in the review and can present the strength of their summary recommendations tied to their assessments of the quality of evidence (e.g., the GRADE system) [19].

### Evaluation of publication bias

Prevention is the best approach to deal with publication bias. In order to minimize publication bias, no language limitation will be imposed during the search strategy. Moreover, if the number of eligible studies exceeds 10, the plot funnel method will be adopted to assess the publication bias. Begg’s and Egger’s methods and a combination of these two will be applied to measure publication bias when the numbers of studies are below 10, over 20, and 10–20, respectively. When *P* values derived from the publication bias assessment are significant (*P* < 0.05), the plot trim and fill method, which is a method based on simulation, will be used to correct the publication bias. This method simulates a number of studies in parts of the funnel plot where no points are scattered. The simulated points will represent missed studies [20]. There will be no publication bias if this process does not transform a strong relationship into a weak one. Otherwise, a high level of publication bias is indicated.

## Discussion

Several studies have assessed the complex association between diabetes and age at menopause. However, such studies did not provide homogeneous findings and in some cases did not distinguish between types of diabetes. For instance, Brand et al. (2015) found that women who developed diabetes at ages under 20 years had a lower age at menopause compared to non-diabetic women. Meanwhile, developing diabetes over 50 years of age delayed the onset of menopause. No relationships were detected between diabetes and age at natural menopause in any other age groups. This study had a large sample size, but did not distinguish between type I and II diabetes [5]. Similarly, another study, which did not differentiate between type I and II diabetes, indicated a positive relationship between the delayed onset of menopause and diabetes. The results of this study suggested late menopause in diabetic women aged 60 years or older [11]. Brand et al. presented evidence that younger women with type II diabetes entered menopause at a younger age and thus had a shorter fertility period. It was also speculated that premature menopause exacerbated the risk of type II diabetes [14]. Sekhar et al. compared age at natural menopause in postmenopausal women with type II diabetes and non-diabetic controls. Their findings revealed an earlier onset of menopause in women with type II diabetes. In other words, type II diabetes decreased the average age of natural menopause [13]. In a recent study, Wellons et al. used an overview design and found that women with type II diabetes were more likely to experience ovulation disorders and earlier menopause [21]. Yarde et al. performed a cross-sectional study to clarify the relationship between type I diabetes and both menopause age and ovarian aging. They calculated the mean age at natural menopause in women with type I diabetes and non-diabetic women as 49.8 ± 4.7 and 49.8 ± 4.2 years, respectively. According to their study, type I diabetes did not exacerbate ovarian aging or decrease age at natural menopause [15]. Apparently, previous studies yielded contradictory results regarding the relationship between type I/II diabetes and age at the onset of menopause. Based on contradictory study results, this protocol was designed to determine the relationship between type I/II diabetes and the age at the onset of menopause.

### Strengths and limitations of this study

While the greatest source of evidence for informed decisions is provided by systematic reviews and meta-analyses, to the best of our knowledge, this will be the first systematic review evaluating the relationship between diabetes and age at menopause.

Authors formally rate or assess the overall body of evidence addressed in the review and can present the strength of their summary recommendations tied to their assessments of the quality of evidence (e.g., the GRADE system).

The absence of studies evaluating the effects of diabetes (DM I/II) on the age at the onset of menopause will be a limitation of this study.
